# Evidence for Complex Interplay between Quorum Sensing and Antibiotic Resistance in Pseudomonas aeruginosa

**DOI:** 10.1128/spectrum.01269-22

**Published:** 2022-10-31

**Authors:** Rakesh Sikdar, Mikael H. Elias

**Affiliations:** a Department of Biochemistry, Molecular Biology and Biophysics, University of Minnesota, Saint Paul, Minnesota, USA; b Biotechnology Institute, University of Minnesota, Saint Paul, Minnesota, USA; University Roma Tre

**Keywords:** *Pseudomonas aeruginosa*, antibiotic resistance, lactonase, quorum sensing

## Abstract

Quorum sensing (QS) is a cell-density-dependent, intercellular communication system mediated by small diffusible signaling molecules. QS regulates a range of bacterial behaviors, including biofilm formation, virulence, drug resistance mechanisms, and antibiotic tolerance. Enzymes capable of degrading signaling molecules can interfere in QS—a process termed as quorum quenching (QQ). Remarkably, previous work reported some cases where enzymatic interference in QS was synergistic to antibiotics against Pseudomonas aeruginosa. The premise of combination therapy is attractive to fight against multidrug-resistant bacteria, yet comprehensive studies are lacking. Here, we evaluate the effects of QS signal disruption on the antibiotic resistance profile of P. aeruginosa by testing 222 antibiotics and antibacterial compounds from 15 different classes. We found compelling evidence that QS signal disruption does indeed affect antibiotic resistance (40% of all tested compounds; 89/222), albeit not always synergistically (not synergistic for 19% of compounds; 43/222). For some tested antibiotics, such as sulfathiazole and trimethoprim, we were able to relate the changes in resistance caused by QS signal disruption to the modulation of the expression of key genes of the folate biosynthetic pathway. Moreover, using a P. aeruginosa-based Caenorhabditis elegans killing model, we confirmed that enzymatic QQ modulates the effects of antibiotics on P. aeruginosa’s pathogenicity *in vivo*. Altogether, these results show that signal disruption has profound and complex effects on the antibiotic resistance profile of P. aeruginosa. This work suggests that combination therapy including QQ and antibiotics should be discussed not globally but, rather, in case-by-case studies.

**IMPORTANCE** Quorum sensing (QS) is a cell-density-dependent communication system used by a wide range of bacteria to coordinate behaviors. Strategies pertaining to the interference in QS are appealing approaches to control microbial behaviors that depend on QS, including virulence and biofilms. Interference in QS was previously reported to be synergistic with antibiotics, yet no systematic assessment exists. Here, we evaluate the potential of combination treatments using the model opportunistic human pathogen Pseudomonas aeruginosa PA14. In this model, collected data demonstrate that QS largely modulates the antibiotic resistance profile of PA14 (for more than 40% of the tested drugs). However, the outcome of combination treatments is synergistic for only 19% of them. This research demonstrates the complex relationship between QS and antibiotic resistance and suggests that combination therapy including QS inhibitors and antibiotics should be discussed not globally but, rather, in case-by-case studies.

## INTRODUCTION

Antibiotic resistance is reported to be rapidly rising, possibly partly due to the overuse of antibiotics in medical, agricultural, and industrial applications (1–3). This risk may have increased during the COVID-19 pandemic with the increased use of antibiotics to prevent secondary bacterial infections in hospitals (4). Pathogenic bacteria that are relevant in human diseases such as Pseudomonas aeruginosa can be resistant to numerous antibiotic treatments (5). It is associated with 10% of nosocomial infections (6) and is the main cause of mortality and morbidity in a debilitating genetic disease such as cystic fibrosis in humans (7). It is listed among the top-priority pathogens by the WHO for immediate research and development (R&D) of new antimicrobials (8). P. aeruginosa shows a remarkable ability to adapt to a wide range of environmental niches due to its high genome plasticity (9, 10). Interestingly, the virulence of P. aeruginosa, like that of many other pathogenic microbes, is regulated by a chemical communication system termed quorum sensing (QS) (11). Consequently, interference in QS signaling is appealing to control microbial pathogens.

Numerous bacteria use QS for communication: they produce, secrete, sense, and respond to small diffusible signaling molecules known as autoinducers (AIs). One main class of autoinducers is autoinducer-I or *N-*acyl homoserine lactones (AHLs). AHL-based QS circuits are reported to regulate the expression of up to 26% of bacterial genes (12) and to modulate bacterial behaviors critical for their pathogenicity, such as virulence factor production, drug resistance, toxin production, motility, and biofilm formation, in a cell-density-dependent manner (13).

P. aeruginosa has three interwoven QS signaling circuits with overlapping genetic targets—namely, LasIR, RhlIR, and Pseudomonas quinolone signal (PQS) in a top-to-bottom order of hierarchy (14, 15). They produce, detect, and respond to autoinducer molecules *N*-3-oxo-dodecanoyl-l-homoserine lactone (3oC12-HSL), *N*-butyryl-l-homoserine lactone (C4-HSL), and alkyl-quinolones, respectively. This sophisticated QS circuitry enables this bacterium to be a versatile and opportunistic pathogen that can adapt to a variety of environmental conditions in the host tissue and to form a robust biofilm that is difficult to disperse (16, 17).

The antibiotic resistance of P. aeruginosa stems from several intrinsic, acquired, and adaptive mechanisms, as elaborated in the references 5, 18, and 19. These mechanisms can be 3-fold: (i) chemical modification of antibiotics using enzymes such as β-lactamases (20), aminoglycoside modifying enzymes (21), and 16s rRNA methylases (22); (ii) modification of biofilm structure (extracellular polymeric substances or exopolysaccharide [EPS]) (23), membrane physiology (outer membrane permeability, lipopolysaccharide [LPS] modification) (24), and/or surface porins (OprF, OprD, and OprH) to reduce antibiotic permeability (25); (iii) expression of multidrug efflux pumps (MexAB-OprM, MexCD-OprJ, MexEF-OprN, and MexXY-OprM) to secrete the antibiotics out of the cell (19); (iv) altering the expression and/or characteristics of genes and proteins targeted by antibiotics (e.g., DNA gyrases, folate biosynthetic pathway genes) (22); (v) utilizing global stress response systems (two-component signaling systems, e.g., PhoPQ, CprRS, ParRS) and phenotypic modifications (swarming and surfing motility, LPS modifications) to adapt to the antibiotic-mediated stress (26–28). Many of these mechanisms for example, surface porins (29), multidrug efflux pumps (30, 31), β-lactam resistance and alginate production (32), biofilm formation and EPS production (33, 34), two-component regulatory systems (35), swarming motility (36), etc., are associated with QS (18, 19, 37), and therefore, antibiotic resistance and QS are possibly interconnected. In support of this hypothesis, some previous studies have highlighted potential synergistic effects between antibiotic treatments and interference in QS (38–45), yet a comprehensive investigation is lacking.

Numerous enzymes capable of hydrolyzing AHLs were isolated and characterized (13, 46). Lactonases, enzymes that hydrolyze and open the lactone ring of AHLs, have been enzymatically and structurally well studied (47, 48). Well-characterized representatives from the phosphotriesterase-like lactonase (PLL) family include VmoLac (49), SisLac (50), PPH (51), or SsoPox (52–54), as well as representatives from the metallo-β-lactamase lactonase (MLL), such as MomL (55), AiiA (56), AaL (57), or GcL (58). By hydrolyzing AHLs, lactonases interfere in QS, a process termed quorum quenching (QQ). QQ enzymes were shown to reduce the virulence of P. aeruginosa both *in vitro* and *in vivo* (55, 59–67). As P. aeruginosa utilizes C4-HSL and 3oC12-HSL-based QS signaling circuits, the AHL preference of the QQ enzyme is important, as it may quench either or both LasIR and RhlIR QS circuits. Moreover, recent studies showed that the substrate specificity of the QQ enzyme affected proteome profiles, virulence factor expression, virulence, and biofilm formation in P. aeruginosa (65, 68).

In this study, we used two QQ lactonases with distinct substrate specificity, SsoPox (52) and GcL (58), to evaluate the effects of AHL signal disruption on the antibiotic resistance profile of the reference strain P. aeruginosa PA14. We observed that signal disruption has complex effects on antibiotic resistance that are dependent on the antibiotic/antimicrobial compound and the QQ enzyme used. We confirmed key observations in independent assays. For the antibiotics sulfathiazole and trimethoprim, we provide evidence that changes in key gene regulation due to interference in QS signaling are responsible for the observed modulation of antibiotic resistance using quantitative reverse transcription PCR (qRT-PCR). Lastly, we demonstrate that these effects on the antibiotic sensitivity of P. aeruginosa can translate *in vivo* in a Caenorhabditis elegans killing assay.

## RESULTS AND DISCUSSION

The effect of QS signal disruption in P. aeruginosa was previously shown to be synergistic with some antibiotics, namely, ciprofloxacin (38–40, 44), ceftazidime (44), and gentamicin (38). However, it remains unclear if this approach can be synergistic with all antibiotic therapies. To address this issue, we investigated if QQ can alter the global antibiotic resistance profile of P. aeruginosa. We used the Biolog Phenotype MicroArrays (69) to characterize the antibiotic resistance profile of P. aeruginosa in the presence of QQ lactonases. While these MicroArrays have been previously used to study the metabolic characteristics of various P. aeruginosa strains under a range of conditions (69–75), and to study chemical resistance in other bacteria (76–78) or mixed microbial communities (79), a comprehensive study of the antibiotic resistance profile of laboratory strains of P. aeruginosa as a function of AHL signaling was not reported to our knowledge.

### Screening with antimicrobials reveals that resistance is modulated by lactonase treatment.

**FIG 1:**
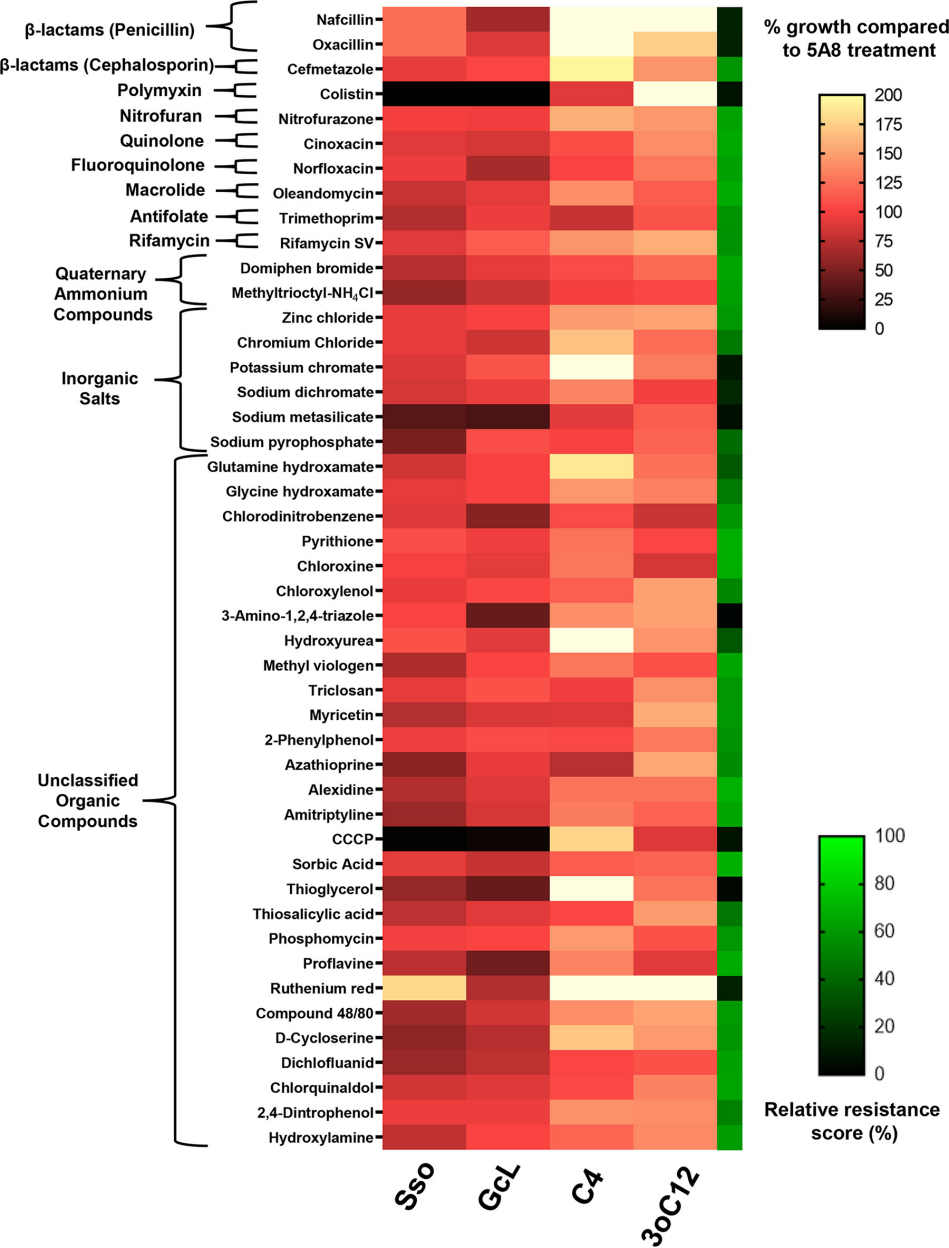
PA14 growth profile for antimicrobials for which lactonase treatment increases sensitivity. Growth of PA14 in the presence of lactonases—SsoPox W263I (Sso) and GcL or exogenously added AHLs—C4-HSL (C4) and 3oC12-HSL (3oC12) is represented as a percentage of PA14 growth in the presence of inactive lactonase SsoPox 5A8 (control) with a white-red-black color scheme. Adjacent to it, an additional heat map strip with a green-black color scheme is overlaid, representing the relative resistance score (see Materials and Methods) on a percentage scale and indicating the sensitivity of PA14 to the tested antibiotics and antibacterial compounds. The tested compounds are grouped according to their classes, indicated on the left. Higher relative resistance scores indicate higher resistance of PA14. All lactonases and AHLs were used at 50-μg/mL and 10-μM final concentrations, respectively.

**FIG 2:**
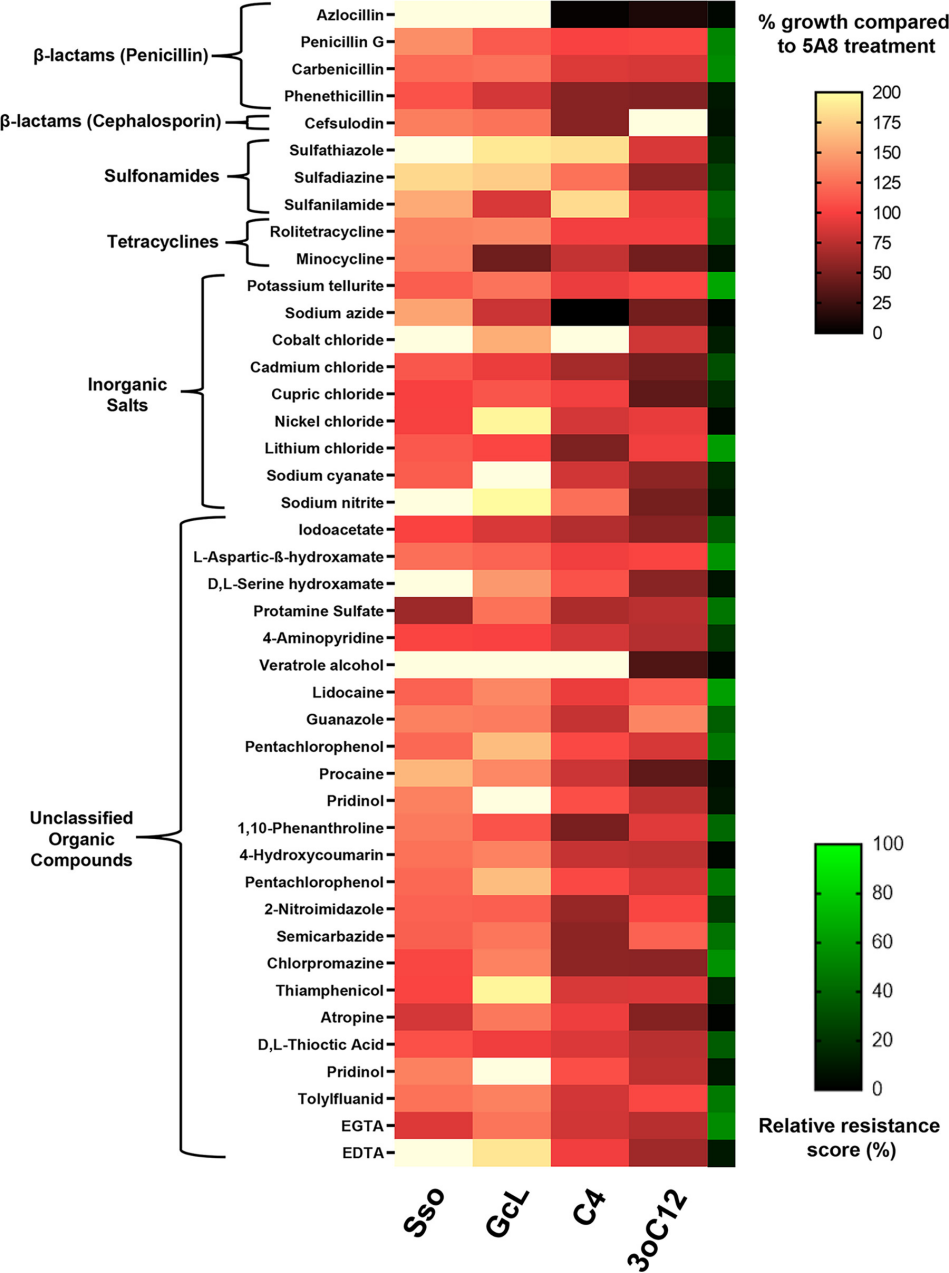
PA14 growth profile for antimicrobials for which lactonase treatment increases resistance. Growth of PA14 in the presence of lactonases—SsoPox W263I (Sso) and GcL or exogenously added AHLs—C4-HSL (C4) and 3oC12-HSL (3oC12) is represented as a percentage of PA14 growth in the presence of inactive lactonase SsoPox 5A8 (control) with a white-red-black color scheme. Adjacent to it, an additional heat map strip with a green-black color scheme is overlaid, representing the relative resistance score (see Materials and Methods) on a percentage scale and indicating the sensitivity of PA14 to the tested antibiotics and antibacterial compounds. The tested compounds are grouped according to their classes, indicated on the left. Higher relative resistance scores indicate higher resistance of PA14. All lactonases and AHLs were used at 50-μg/mL and 10-μM final concentrations, respectively.

We quantified the growth of PA14 using 10 Phenotype MicroArrays (Biolog PM11 to PM20) containing a total of 222 unique antibiotics and antibacterial compounds in the presence of 4 experimental treatments—two lactonases (SsoPox W263I, GcL) and two pure exogenously added AHLs (C4-HSL, 3oC12-HSL)—and 1 control treatment, the inactive lactonase SsoPox 5A8 mutant (Fig. 1 and 2; see Fig. S1 to S5 in the supplemental material). An increase in the growth of PA14 in the presence of antimicrobial compounds relates to an increase in resistance (or a decrease in sensitivity) against these compounds. Conversely, decreased growth relates to an increase in sensitivity (or a decrease in resistance) against these compounds. It is in this context that the terms “sensitivity” and “resistance” are used throughout this article.

Growth patterns of PA14 in the presence of antibiotics or antibacterial compounds appear considerably altered with lactonases/AHLs, and these alterations are also a function of the concentrations of these compounds (Fig. S1 to S5). As expected for most of the antibiotics and antibacterial compounds, the growth of PA14 decreased with the increasing concentration of the compounds. The whole screening results shown in Fig. S1 to S5 were reduced using a set of criteria described in Materials and Methods to focus on the most robust changes upon treatments. Compounds of particular interest are shown in Fig. 1 and 2.

**FIG 3:**
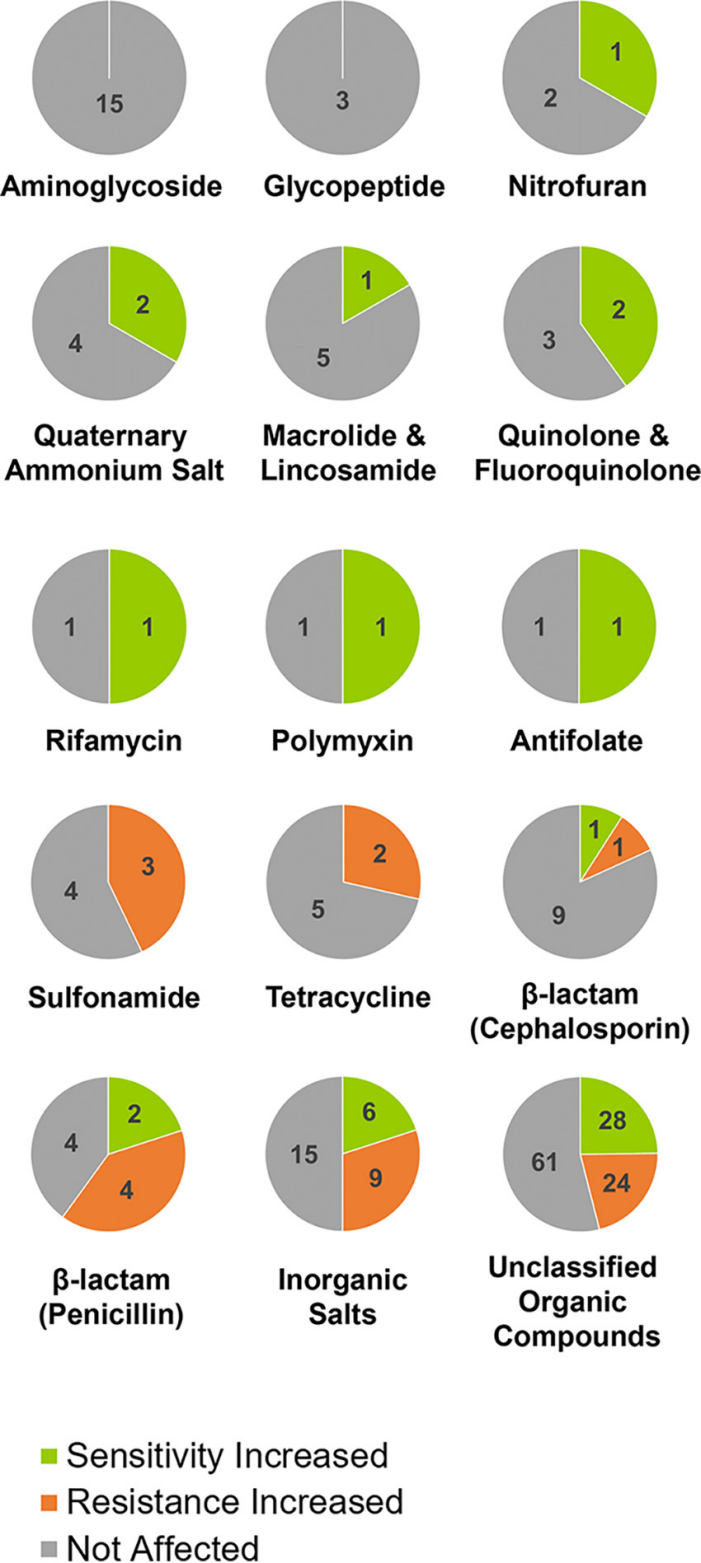
Effects of AHL signal disruption on different classes of antimicrobials. Pie charts showing the fraction of tested compounds from different classes for which a change in sensitivity and/or resistance of PA14 upon AHL signal disruption was observed.

Results shown in Fig. 1 and 2 and Fig. S1 to S5 show that the antibiotic sensitivity profile of PA14 is dependent on QS signaling. Indeed, these tested antibiotics and antibacterial compounds can be broadly classified into two major groups. The first group (Fig. 1) includes conditions where lactonase treatment suppresses and/or exogenous AHLs promote the growth of PA14. QQ increases the sensitivity (or decreases the resistance) of PA14 against this group of molecules. The second group of tested antibiotics and biocides shows the opposite trend (Fig. 2): for these molecules, the lactonase treatments promote and/or exogenous AHLs suppress the growth of PA14, suggesting that QQ decreases the sensitivity (or increases the resistance) of PA14. As expected, changes in sensitivity of PA14 are more easily observed at antibiotic concentrations that exhibited a lower relative resistance score (Fig. S1 to S5), usually below 70%. The changes in sensitivity/resistance of PA14 to different groups of antimicrobial compounds are summarized in pie charts (Fig. 3) and discussed later in the manuscript.

Among the tested compounds that did not reduce PA14 growth much (resistance score, >70%), a change in growth upon treatment (>25% in at least one treatment compared to the 5A8 control) was observed for 10 molecules (Fig. S6). The sensitivity of PA14 to all these 10 compounds was significantly increased by QQ lactonase treatment, compared to the control. For example, in the presence of chlorhexidine, PA14 showed a 19% and 42% reduction in growth upon QQ with SsoPox W263I and GcL, respectively, and a 9% and 11% increase in growth upon the addition of C4-HSL and 3oC12-HSL, respectively, compared to the SsoPox 5A8-treated control. These types of observations are likely outliers and are largely eliminated using our filtering criteria (discussed in Materials and Methods).

We used the lactonases SsoPox W263I and GcL for AHL signal disruption. These enzymes were enzymatically and structurally characterized in previous studies (52–54, 58, 68). Specifically, SsoPox shows a strong AHL preference for long acyl chain AHL substrates (>C8) and low activity against C4-HSL. On the other hand, GcL exhibits a broad substrate preference and hydrolyzes both C4-HSL and 3oC12-HSL with high proficiency. The difference in kinetic properties of these two enzymes was previously described (68). Since P. aeruginosa simultaneously utilizes two QS circuits based on C4-HSL and 3oC12 HSL, these two lactonases with distinct specificities may differentially affect these circuits. Differential QQ with these enzymes was previously described and resulted in differential proteome profiles, virulence factor expression, virulence, and biofilm formation in P. aeruginosa (65, 68).

To assess whether changes in antibiotic resistance would be sensitive to the differential quenching of AHL QS circuits, we focused on compounds for which robust resistance changes can be observed (see Materials and Methods) and for which SsoPox W263I and GcL treatments show large differences (Fig. S7). For 27% of all tested compounds (60/222), changes in resistance upon lactonase treatment are unidirectional, i.e., both lactonases either increase (32/60) or decrease (28/60) growth compared to the control. Within these groups, some differences can be observed. For example, for a fraction of the compounds (5% of all tested compounds; 11/222), SsoPox W263I treatment resulted in >25 percentage points higher PA14 growth increase than GcL treatment (Fig. S7, top heatmap panel). For another fraction (4.5% of all tested compounds; 10/222), GcL treatment yielded >25 percentage points higher PA14 growth than SsoPox W263I (Fig. S7, middle heatmap panel). For another group of compounds (5.4% of all tested compounds; 12/222), treatment with both lactonases resulted in opposite changes in resistance, i.e., the change in the growth of PA14 was opposite with SsoPox W263I and GcL by >25% for at least one lactonase compared to the 5A8-treated control (Fig. S7, bottom heatmap panel). These observations suggest that the AHL-dependent changes in the resistance profile of PA14 may be sensitive to the AHL substrate specificity of the QQ lactonase.

### Replication experiments show that interference in AHL signaling induces changes in resistance that are variable in sign and magnitude.

**FIG 4:**
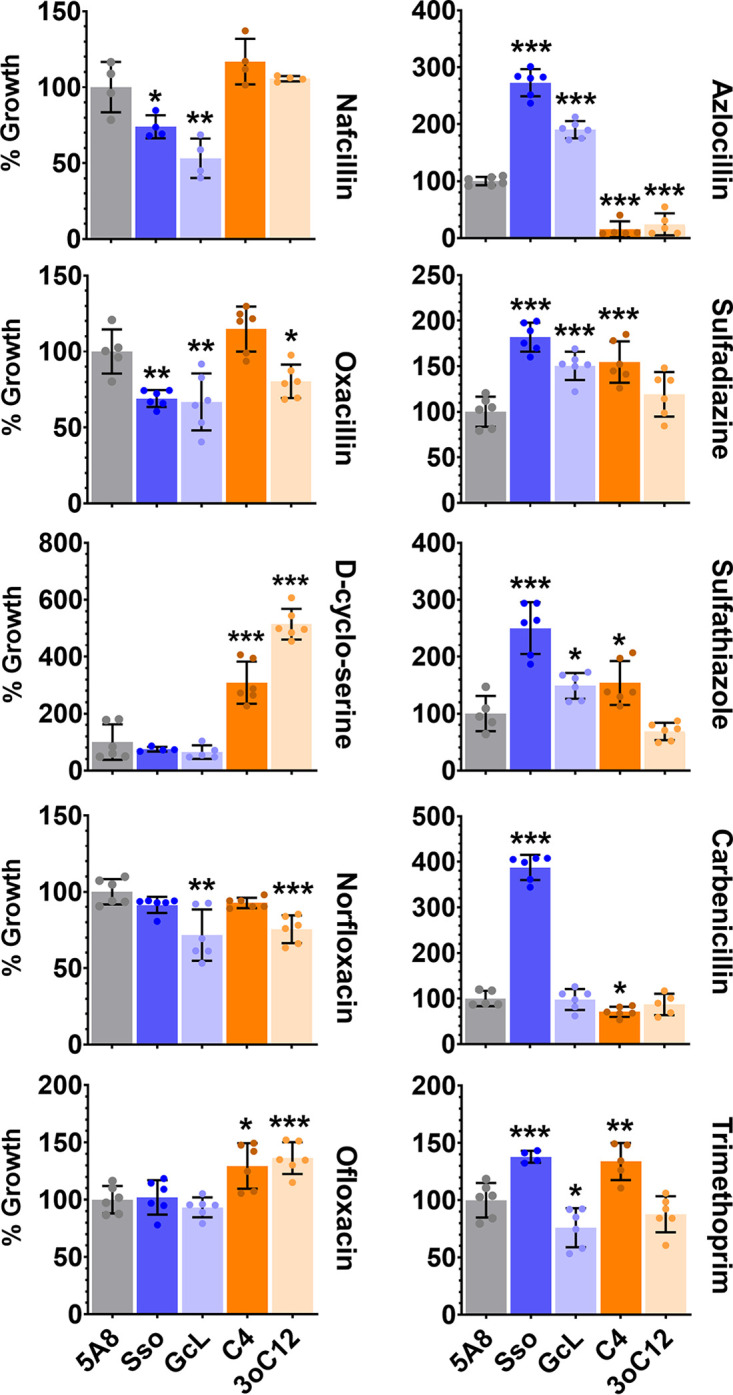
Replicated PA14 growth experiments in the presence of 10 antimicrobials identified in the screening experiment and the treatments. Treatments are QQ lactonases—SsoPox W263I (Sso) and GcL or exogenously added AHLs—C4-HSL (C4) and 3oC12-HSL (3oC12), compared to the control treatment (inactive lactonase SsoPox 5A8 [5A8]). PA14 growth in all treatments was normalized to the respective 5A8 control. All lactonases and AHLs were used at 50-μg/mL and 10-μM final concentrations, respectively. The following concentration of antibiotics were used: 200 μg/mL nafcillin, 800 μg/mL oxacillin, 180 μg/mL d-cyclo-serine, 0.25 μg/mL norfloxacin, 0.5 μg/mL ofloxacin, 5 μg/mL azlocillin, 25 μg/mL sulfadiazine, 25 μg/mL sulfathiazole, 50 μg/mL carbenicillin, and 20 μg/mL trimethoprim. All experiments were done and all data are represented as the mean and standard deviation of at least quadruplicate experiments. The statistical significance of all treatments compared to the control (5A8) was calculated using unpaired two-tailed *t* tests with Welch’s correction, and significance values are indicated as ***, *P* < 0.0005; **, *P* < 0.005; and *, *P* < 0.05.

Observations derived from the experiments with the Biolog Phenotype MicroArrays were replicated for some key candidate compounds. First, dose-response experiments against PA14 were performed (Fig. S8) and used to determine their sublethal concentration values. This was needed because of the proprietary conditions of Biolog Phenotype MicroArrays. The results (Fig. 4) confirm that the antibiotic resistance profile of PA14 is dependent on AHL signaling. Similar to the screening experiment, the same two groups of compounds can be established: (i) antibiotics for which lactonases suppress and/or exogenous AHLs promote the growth of PA14 (e.g., nafcillin, oxacillin, d-cyclo-serine, norfloxacin, and ofloxacin) and (ii) antibiotics for which lactonases promote and/or exogenous AHLs suppress the growth of PA14 (e.g., azlocillin, sulfadiazine sulfathiazole, and carbenicillin). Lactonase treatment increased the sensitivity of PA14 against nafcillin, oxacillin, and norfloxacin by up to 46%, 33%, and 28% respectively, compared to the control. In contrast, lactonase treatment increased the resistance of PA14 against azlocillin, sulfadiazine, sulfathiazole, and carbenicillin by up to 173%, 82%, 150%, and 288%, respectively, compared to the control. In the case of d-cyclo-serine and ofloxacin, no significant effect of lactonase treatment on PA14 sensitivity was observed. However, the addition of exogenous AHLs to stimulate QS increased the resistance of PA14 to d-cyclo-serine and ofloxacin by up to 414% and 36%, respectively, compared to the control. These results suggest a complex relationship between QS and antibiotic resistance in PA14. For example, the addition of C4-HSL, similar to lactonase treatments, increased the resistance of PA14 to antibiotics such as sulfadiazine, sulfathiazole, and trimethoprim, by 55%, 54% and 34%, respectively, compared to the control (Fig. 4). This is not the case with 3oC12-HSL treatment, for which resistance of PA14 does not differ from that of control. Treatment with GcL increased the sensitivity and with C4-HSL increased the resistance of PA14 against trimethoprim by 24% and 34%, respectively, compared to the control (Fig. 4). This is consistent with the lactonase substrate specificity, because GcL degrades C4-HSL proficiently (and SsoPox W263I does not); therefore, GcL and the addition of C4-HSL appear to show antagonistic effects.

For some compounds, including procaine and coumarin, the results are surprising: both lactonase and AHL treatments increase the sensitivity of PA14 against these compounds by up to 48% and 35%, respectively (Fig. S11). Coumarin was previously reported to be an inhibitor of QS circuits and additional signaling pathways in P. aeruginosa (80, 81), and this is expected to affect the results of our experiments. In the case of procaine, it was previously shown to enhance antibiotic resistance in P. aeruginosa by increasing the expression of the MexCD-OprJ and MexAB-OprM efflux pumps (82) and is therefore also likely to affect the outcome of our experiments.

Overall, most observations from these replicated experiments are consistent with results from the screening (Fig. S9 to S11). However, some differences can be noted for a few compounds (e.g., ofloxacin, procaine, carbonyl cyanide *m*-chlorophenylhydrazone [CCCP], colistin, and trimethoprim). These discrepancies likely originate from the sensitivity of QS-dependent regulations of growth conditions. The latter could not be exactly reproduced due to the proprietary composition of Biolog conditions. The fact that QS-dependent regulations are highly sensitive to growth conditions and treatment is illustrated by two different sublethal concentrations (150 and 180 μg/mL) of d-cyclo-serine (Fig. S12). While lactonase treatments did not alter the sensitivity of PA14 to 150 μg/mL d-cyclo-serine, AHL treatments increased its resistance to the compound by 73%. When the concentration of d-cyclo-serine was increased to 180 μg/mL, AHL treatments increased the resistance of PA14 to the compound by up to 414%. Additionally, we note that this increased resistance may originate from the positive regulation of alanine racemase in P. aeruginosa by QS, a target of d-cyclo-serine (see Table S1 in reference 83 for studies conducted with strain PAO1).

**FIG 5:**
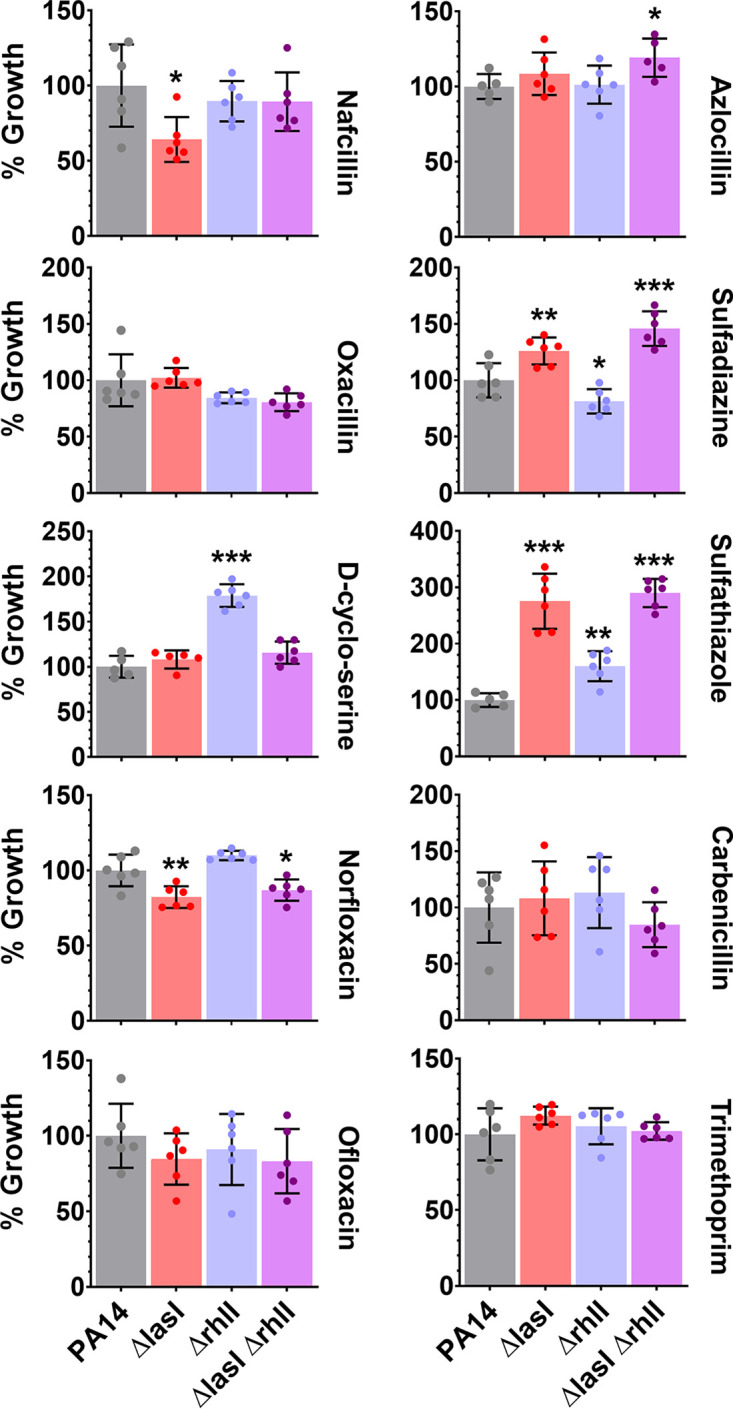
Growth comparisons between PA14 and its AHL synthase-deficient mutants in the presence of 10 antimicrobials identified in the screening experiment and interference in quorum sensing compounds. Growth of the PA14 wild type is compared to its Δ*lasI* and Δ*rhlI* single and double deletion mutants in the presence of the following concentration of antibiotics: 200 μg/mL nafcillin, 800 μg/mL oxacillin, 180 μg/mL d-cyclo-serine, 0.25 μg/mL norfloxacin, 0.5 μg/mL ofloxacin, 5 μg/mL azlocillin, 25 μg/mL sulfadiazine, 25 μg/mL sulfathiazole, 50 μg/mL carbenicillin, and 20 μg/mL trimethoprim. All experiments were done and all data are represented as the mean and standard deviation of at least four replicate experiments. The statistical significance of all treatments compared to the control (5A8) was calculated using unpaired two-tailed *t* tests with Welch’s correction, and significance values are indicated as ***, *P* < 0.0005; **, *P* < 0.005; and *, *P* < 0.05.

In order to confirm the importance of QS in antimicrobial resistance of PA14, we used AHL-synthase-deficient mutants of PA14 that do not produce C4-HSL and/or 3oC12-HSL. We compared the resistance of P. aeruginosa PA14 and its mutants Δ*lasI* (SM51; does not produce 3oC12-HSL), Δ*rhlI* (SM52; does not produce C4-HSL), and Δ*lasI* Δ*rhlI* (SM53; does not produce C4-HSL or 3oC12-HSL). For all antimicrobials except oxacillin, carbenicillin, and trimethoprim, we observed similar patterns in changes in antimicrobial resistance of PA14 when comparing lactonase treatment (Fig. 4) and AHL-synthase mutants (Fig. 5). However, there are considerable differences in the magnitude, if not the direction, of observed changes in antimicrobial resistance with these two different approaches. For example, the AHL synthase mutants show increased sensitivity to nafcillin and norfloxacin (by up to 36% and 18%, respectively; Fig. 5). This is similar to the changes observed with lactonase treatment (up to 46% and 28%, respectively; Fig. 4). A similar pattern in resistance increase is also observed with azlocillin, sulfadiazine, and sulfathiazole. The AHL synthase mutants exhibit increased resistance to these compounds (by up to 19%, 46%, and 190%, respectively; Fig. 5). This is consistent with changes in antibiotic resistance observed with lactonase treatment (up to 173%, 82%, and 150% with azlocillin, sulfadiazine, and sulfathiazole, respectively; Fig. 4). These changes in PA14’s resistance to azlocillin, sulfadiazine, and sulfathiazole also varied between the different tested AHL-synthase mutants (Fig. 5). In the case of the d-cyclo-serine treatment, only the lack of C4-HSL (Δ*rhlI*) increased the resistance of PA14 to this compound by 79%. As neither SsoPox nor GcL exclusively degrade C4-HSL, it is very difficult to compare this observation with lactonase treatments. There were no changes in d-cyclo-serine resistance observed in PA14 when either 3oC12-HSL or both AHLs were absent (Fig. 5), consistent with lactonase treatments (Fig. 4). Similarly, for ofloxacin, lactonase treatment and QS mutants showed no changes in sensitivity of PA14 (Fig. 4 and 5). However, no effects on oxacillin, carbenicillin, and trimethoprim sensitivity were observed for the tested QS mutants (Fig. 5), unlike with lactonase treatments (Fig. 4). This discrepancy may suggest that other mechanisms affected the mutations, or the lactonase treatments may affect antimicrobial resistance of PA14. Overall, the high consistency between lactonase treatments and QS mutants confirms the importance of QS in the antibiotic resistance profile in PA14.

While PA14 is one of the reference P. aeruginosa strains, the observed changes in antimicrobial resistance may differ in other isolates. We therefore used a multidrug resistant (MDR) strain of P. aeruginosa, specifically, a levofloxacin-resistant strain designated CI19. Indeed, CI19 was found to be resistant to a multitude of antibiotics (Fig. S13) at concentrations that were determined to be sublethal for PA14 (Fig. S8). We tested the effect of lactonases and AHLs on the nafcillin, sulfathiazole, and norfloxacin resistance of CI19 at dosages that were determined to be sublethal for PA14. As shown in Fig. S14, lactonase treatment increased the sensitivity of CI19 to nafcillin by up to 28%, while boosting QS by adding exogenous 3oC12-HSL increased the nafcillin resistance of CI19 by 28%. This pattern is similar to what was observed for PA14 (Fig. 4). However, unlike PA14, both the sulfathiazole and norfloxacin resistance of CI19 was reduced by up to 31% and 28%, respectively, upon either lactonase or AHL treatment. This observation may be due to the resistance of CI19 to sulfathiazole and norfloxacin at the concentrations used in these experiments, in contrast to PA14 (Fig. S13). Indeed, the tested antibiotic concentration appears critical, as seen previously (Fig. S12). Additionally, while LasR may be present in CI19 (65), the status of QS circuits and the nature of AHL molecules produced by CI19 are unknown. Overall, this confirms that QS and QQ strategies can affect antibiotic resistance in MDR strains of P. aeruginosa.

Altogether, these experiments (Fig. 1–5; Fig. S1 to S14) show that (i) AHL-mediated QS signaling can significantly alter the antibiotic resistance profile of P. aeruginosa and (ii) changes in resistance are variable in sign and magnitude in ways that are not inferable from the properties of the tested antibiotics and antibacterial compounds.

### The sign of the effect of lactonase treatment on resistance varies for the different tested antimicrobial groups.

We classified the tested antibiotics and antibacterial compounds according to their type, class, or mode of action—namely, aminoglycoside, antifolate, β-lactam cephalosporin, β-lactam penicillin, quinolone and fluoroquinolone, glycopeptide, macrolide and lincosamide, nitrofuran, polymyxin, quaternary ammonium salt, rifamycin, sulfonamide, tetracycline, and inorganic salt. Antibacterial compounds not fitting into any of these categories were labeled unclassified organic compounds. Because the bacterial resistance mechanisms developed are often conserved and specific to certain antibiotic or antibacterial compounds, we hypothesized that the observed changes may be shared by compound classes. This is corroborated by previous observations showing that QQ enzymes can alter the levels of proteins typically involved in antibiotic resistance in PA14 proteomics studies (68).

Consistently, we observe groups of molecules for which QQ lactonase treatment is either neutral or increases the sensitivity of PA14 (Fig. 3; i.e., antifolates, polymyxins, rifamycins, quinolones, fluoroquinolones, nitrofurans, macrolides, lincosamides, and quaternary ammonium compounds). We also observe groups of molecules for which QQ lactonase treatment is either neutral or decreases the sensitivity of PA14 (Fig. 3; sulfonamides, tetracyclines). Other groups, for which QS modulation can either increase or decrease resistance, include inorganic salts, an unclassified group of organic compounds, and the β-lactam group (penicillins and cephalosporins). We note that for most conditions, QS alteration resulted in no change in PA14 resistance. This may be caused by the fact that the involved pathways are unaffected by quorum sensing, and/or the inherent resistance of PA14 against these compounds, and the fact that the tested concentrations were too low to sufficiently challenge PA14 growth. This is illustrated by the observed correlation between the relative resistance score and the magnitude of the observed modulations by QS alterations.

AHL signal disruption by lactonases led to exclusively increased sensitivity of PA14 to 40% of tested fluoroquinolones (2/5) on the Biolog Phenotype MicroArrays (Fig. 3). Fluoroquinolone antibiotics inhibit bacterial DNA synthesis by targeting DNA topoisomerases essential for DNA replication in bacteria—DNA gyrase (in Gram-negative bacteria) and topoisomerase IV (in Gram-positive bacteria) (84). Typical mechanisms of resistance against this class of antibiotics involve antagonistic mutations in target topoisomerases, use of efflux pumps to secrete the drugs out of the cell, or reducing their membrane permeability through porins (85). In the case of P. aeruginosa, the RND efflux pumps MexAB-OprM (86), MexCD-OprJ (87), and MexEF-OprN (88) were implicated in fluoroquinolone resistance. Here, the increased sensitivity of PA14 observed with lactonase treatment might be related to decreased OprM levels upon lactonase treatment as was previously reported (68). Conversely, adding exogenous AHLs increases resistance to ofloxacin (Fig. 4). This is consistent with a previous report of a LasR overproducing P. aeruginosa strain found to be more resistant to ofloxacin, and in the absence of LasR (QS compromised), the overexpression of master regulator RpoS can restore the loss of ofloxacin resistance (89). We also observe that lactonase treatment with GcL (but not SsoPox) increased its sensitivity toward norfloxacin (Fig. 4). This resonates with other studies reporting that the loss of function of the regulator protein NfxB, which represses the production of the MexCD-OprJ efflux pump, is associated with increased resistance of P. aeruginosa against norfloxacin (90, 91) and is under the regulatory control of the master regulator VqsM, which promotes QS by producing LasI (an enzyme that synthesizes 3oC12-HSL in PA14) (92). Additionally, lactonase-mediated QQ also had a synergistic effect when coadministered with the fluoroquinolone antibiotic ciprofloxacin and prevented the spread of P. aeruginosa in a burn wound infection model (39).

For other groups, such as β-lactams, alteration of QS modulation can either increase or decrease resistance. When AHL signaling is inhibited by lactonases, P. aeruginosa becomes more sensitive to nafcillin and oxacillin but more resistant to azlocillin and carbenicillin (Fig. 4). This discrepancy is possibly a result of the use of different resistance mechanisms against these drugs. While P. aeruginosa strains were reported to produce β-lactamases such as AmpC (93) and OXA-50 (20) that offer protection against a broad range of β-lactam antibiotics, the resistance against some classes of β-lactam antibiotics can be mediated by the regulation of the expression of outer membrane porins and efflux pumps such as OprD and MexXY-OprM (94). In support of this hypothesis, a previous proteomics study reported an increase in OprD and a decrease in OprM levels in PA14 upon AHL signal disruption using lactonases SsoPox W263I and GcL (68). A synergy between nafcillin and QQ agents was also previously reported for Gram-positive pathogens—Staphylococcus aureus (95) and Staphylococcus epidermidis (96).

### AHL-mediated quorum sensing and resistance of P. aeruginosa to sulfathiazole and trimethoprim.

Sulfathiazole and trimethoprim target the enzymes involved in the biosynthesis of folates in bacteria (97). Sulfathiazole belongs to a class of compounds called sulfonamides that act as competitive inhibitors of the enzyme dihydropteroate synthase (DHPS; encoded by the *folP* gene in PA14). Trimethoprim, an antifolate compound, targets the enzyme dihydrofolate reductase (DHFR; encoded by the *folA* gene in PA14) (98). While DHPS acts upstream of the folate biosynthesis pathway converting *p*-amino benzoic acid to dihydropteroate, DHFR is the enzyme catalyzing the terminal step of the pathway leading to the reduction of dihydrofolate to tetrahydrofolate (99). Bacteria can develop resistance against sulfonamides or trimethoprim by using several mechanisms (100, 101): accumulation of compensatory mutations in the native DHPS/DHFR enzymes that prevent binding of the drugs (102, 103), use of drug efflux pumps and altering membrane barrier permeability (104, 105), horizontal acquisition of foreign drug-resistant DHPS/DHFR variants or homologs either on the chromosome via mobile genetic elements or via plasmids (106, 107), genetic regulation of DHPS/DHFR production (108, 109), or production of specialized enzymes that cleave these drugs (110).

**FIG 6:**
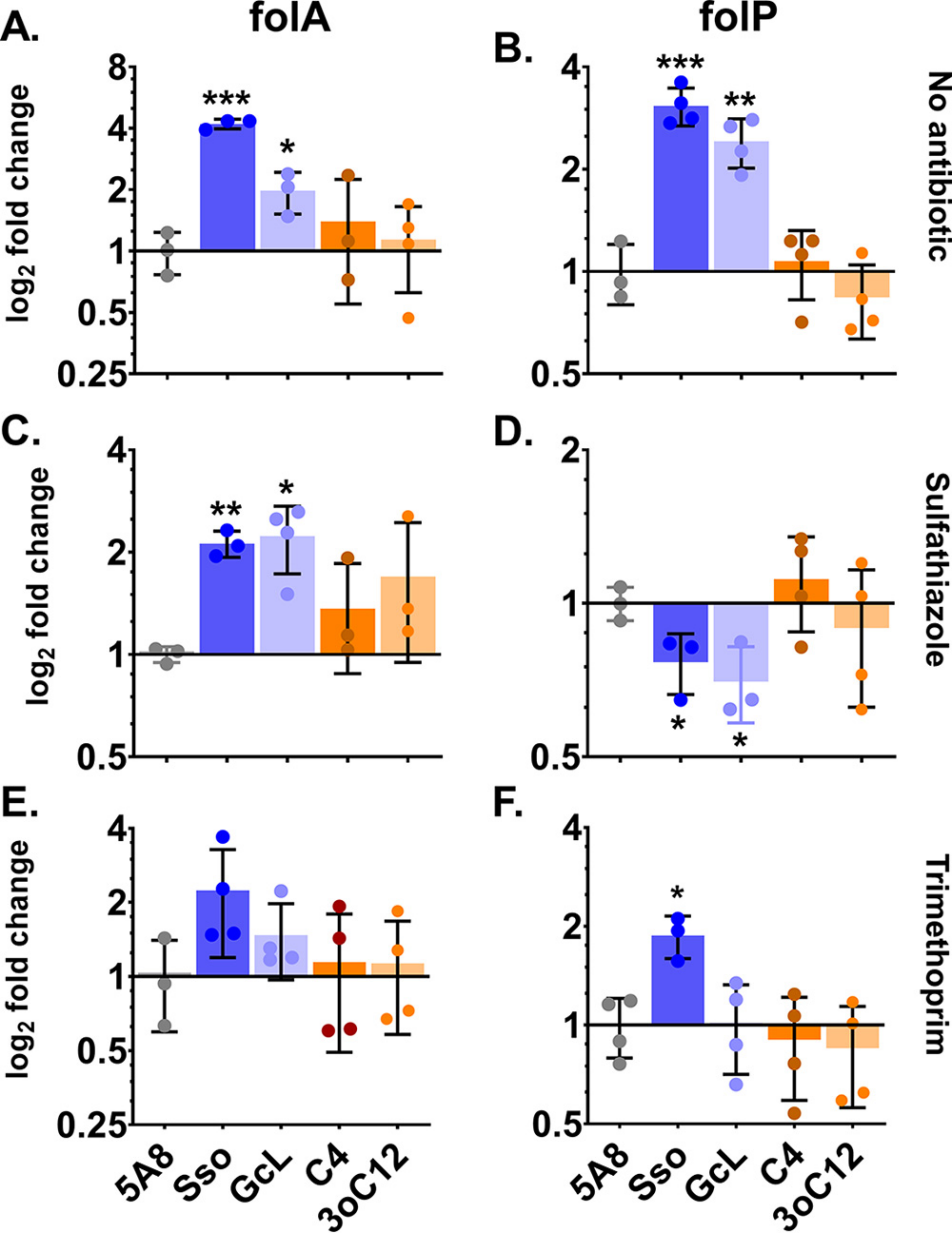
Changes in mRNA levels (as measured by qRT-PCR) of genes *folA* and *folP* with sulfathiazole and trimethoprim and as a function of quorum sensing interference. (A to F) The treatments used are no antibiotics (A, B) or with the antibiotics sulfathiazole (C, D) and trimethoprim (E, F). The tested conditions include the QQ lactonases, SsoPox W263I (Sso) and GcL, or exogenously added AHLs, C4-HSL (C4) and 3oC12-HSL (3oC12), compared to the control treatment (inactive lactonase SsoPox 5A8 [5A8]). *folA* and *folP* mRNA levels in all treatments were determined using the relative quantification method using the *recA* gene as the endogenous control and normalized to the respective 5A8 control, which is set to 1 on a log_2_ scale. All lactonases and AHLs were used at 50 μg/mL and 10 μM final concentrations, respectively. The following concentration of antibiotics was used: 25 μg/mL sulfathiazole and 20 μg/mL trimethoprim. All experiments were done and all data are represented as the mean and standard deviation of at least triplicates. The statistical significance of all treatments compared to the control (5A8) was calculated using unpaired two-tailed *t* tests with Welch’s correction, and significance values are indicated as ***, *P* < 0.0005; **, *P* < 0.005; and *, *P* < 0.05.

In this study, the modulations of the resistance to sulfonamide and trimethoprim observed in P. aeruginosa PA14 by QS are likely to originate from the genetic regulation of *folA* and *folP* genes. To test this hypothesis, we carried out qRT-PCR experiments to determine the expression levels of *folA* and *folP* transcript mRNA under a variety of conditions (Fig. 6). In the absence of any antibiotics, when QS is attenuated by lactonases, *folA* (Fig. 6A) and *folP* (Fig. 6B) genes are upregulated by 4-fold and 3-fold, respectively, compared to an inactive enzyme-treated control. This suggests a possible increased metabolic demand for folate when QS is suppressed. When AHL signaling is attenuated and PA14 is challenged with sulfathiazole, expression of *folA* remains significantly upregulated (from 2- to 2.5-fold; Fig. 6C) compared to the inactive enzyme-treated control. However, *folP* expression is downregulated by 30% (Fig. 6D). In the presence of trimethoprim, only *folP* expression is increased (2-fold, Fig. 6F) compared to control. This downregulation of *folP* upon AHL signal disruption is also observed in the absolute levels of transcripts (Fig. S15). Under most conditions (Fig. S15), as expected, competitive inhibitors such as sulfathiazole and trimethoprim increase the expression of *folA* and *folP* genes compared to the no-antibiotic control. However, when AHL signaling is disrupted by lactonases, the absolute *folP* levels in sulfathiazole-treated PA14 are reduced to levels similar to those observed for untreated PA14.

These observations suggest that QS regulates the expression of *folP* and *folA* genes in PA14. We also observe that QQ leads to increased resistance of PA14 to sulfathiazole (Fig. 6). In a study previously conducted in yeast auxotrophs (108), sulfonamides, in addition to inhibiting DHPS, are metabolized to sulfa-dihydropteroate, a compound that inhibits the downstream enzyme DHFR and, thereby, the biosynthesis of folate. This inhibition can be thwarted by overproducing DHFR. Conducting a challenge with sulfathiazole and AHL signal disruption, we observed a simultaneous downregulation of *folP* (DHPS; Fig. 6D) and overproduction of *folA* (DHFR; Fig. 6C). This *folP* and *folA* regulation might explain the higher resistance of PA14 to sulfathiazole when QS is disrupted.

Unlike sulfathiazole, changes in resistance of PA14 against trimethoprim upon AHL signal disruption cannot be explained solely by the observed changes in *folP* and *folA* regulation. An increase in absolute (Fig. S15) and relative levels (compared to inactive lactonase 5A8 treatment; Fig. 6F) of *folP* in the presence of trimethoprim and lactonase can be observed, and it might contribute to the observed increased resistance (Fig. 4). Adaptive resistance mechanisms such as the regulation of efflux pumps might be involved in the QS-dependent regulation of trimethoprim resistance in PA14. For example, MexEF-OprN efflux pump overexpression (due to loss of function of its repressor NfxC) renders P. aeruginosa resistant to several antibiotics, including trimethoprim (111). Similarly, the BpeEF-OprC efflux pump system confers trimethoprim resistance to Burkholderia pseudomallei (112).

### AHL signaling can modulate the potency of antibiotic treatments in a P. aeruginosa C. elegans infection model.

Upon observing that interference in AHL signaling modulates antibiotic resistance of P. aeruginosa in liquid cultures, we examined whether these modulations would translate into altered PA14 pathogenicity in an *in vivo* Caenorhabditis elegans infection model. C. elegans is a nematode whose genotypes and phenotypes have been characterized and that has long served as a model organism in cellular, molecular, and developmental biology research (113, 114). It has also been extensively used as a convenient model animal host in a plethora of microbial infection assays (115–118). We adapted the standard liquid killing assay (see Materials and Methods). Lactonases were previously shown to reduce the virulence of P. aeruginosa against C. elegans in infection assays (55).

**FIG 7:**
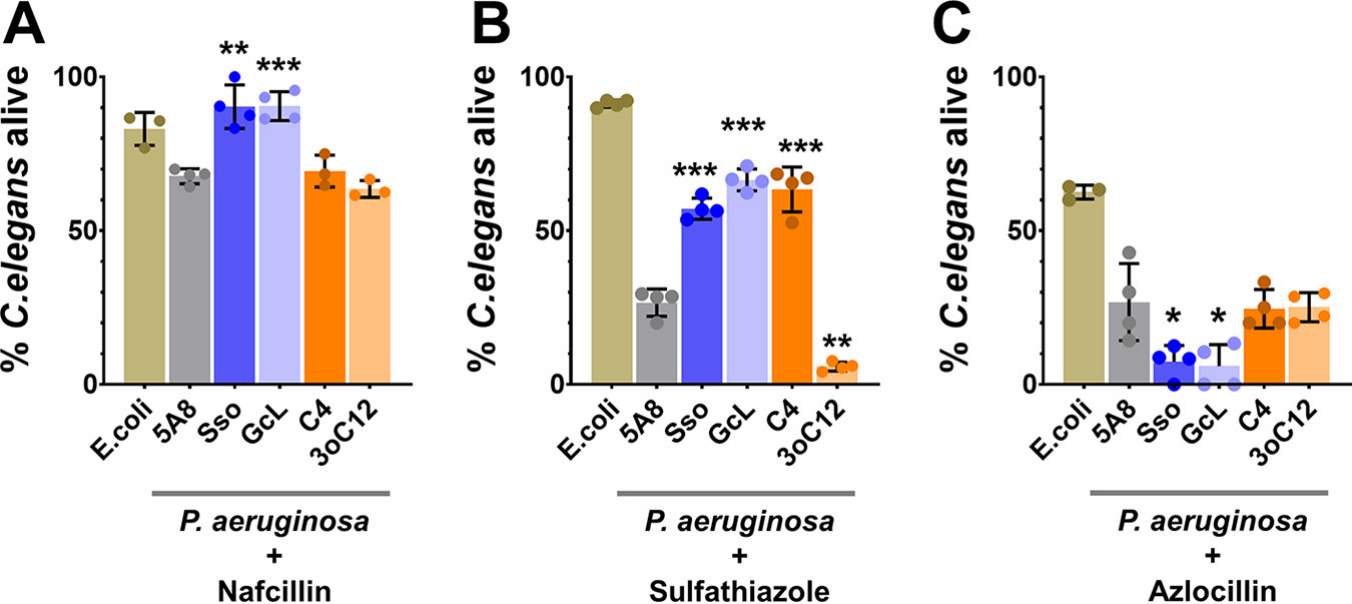
*In vivo* C. elegans infection to evaluate the effects of the interference in quorum sensing and antibiotic treatments. (A to C) Assays were performed with the antibiotics nafcillin (A), sulfathiazole (B), and azlocillin (C) upon treatment with QQ lactonases, SsoPox W263I (Sso) and GcL, or exogenously added AHLs, C4-HSL (C4) and 3oC12-HSL (3oC12), compared to the control treatment (inactive lactonase SsoPox 5A8 [5A8]). The virulence of PA14 is exemplified by the death of C. elegans upon infection. Mortality is represented as the percentage of nematodes that survived the assay. E. coli strain OP50 was used as a nonvirulent control for the assays. All lactonases and AHLs were used at 100 μg/mL and 20 μM final concentrations, respectively. The concentrations of antibiotics were 200 μg/mL nafcillin, 25 μg/mL sulfathiazole, and 5 μg/mL azlocillin. All experiments were done and all data are represented as the mean and standard deviation of at least triplicates. The statistical significance of all treatments compared to the control (5A8) was calculated using unpaired two-tailed *t* tests with Welch’s correction, and significance values are indicated as ***, *P* < 0.0005; **, *P* < 0.005; and *, *P* < 0.05.

When challenged with nafcillin or sulfathiazole, the pathogenicity of PA14 in a C. elegans killing assay is significantly modulated by AHL-based quorum sensing (Fig. 7A and B). For instance, in the presence of nafcillin (Fig. 7A) and sulfathiazole (Fig. 7B), QQ lactonase treatment reduced the virulence of PA14 against C. elegans. Nematode survival increased by up to 34% and 150% for nafcillin and sulfathiazole, respectively, upon treatment with SsoPox W263I and GcL compared to the inactive enzyme control. Conversely, with azlocillin, nematode mortality is increased by lactonase treatment by up to 78% compared to control (Fig. 7C).

Alteration of the antibiotic resistance profile of PA14 *in vivo* by AHL-based QS is further evidenced by the observed modulations of nematode mortality upon treatment with AHL signaling molecules. For example, treatment of PA14 with sulfathiazole and 3oC12-HSL caused an increase in nematode mortality (+78%), whereas sulfathiazole and C4-HSL treatment led to an increase in survival (+138%) compared to an inactive lactonase-treated control. This may suggest that the regulation of virulence, in the presence of sulfathiazole, is similar to that described for pyocyanin production. Indeed, Rhl QS circuit agonists like C4-HSL were reported to suppress pyocyanin production in P. aeruginosa in a PQS-dependent manner (119, 120).

QS signaling and corresponding pathogenicity of P. aeruginosa can be subjected to translation-level thermoregulation by molecular factors such as RNA thermometers (121). Higher growth temperatures can enhance QS signaling circuits and boost virulence factor production. Therefore, it is of interest to verify that quorum quenching affects antibiotic resistance *in vitro* and *in vivo* at the same temperature. To do this, we determined the effect of lactonases and AHLs on the nafcillin, azlocillin, and sulfathiazole sensitivity of PA14 at a lower temperature (26°C), close to the *in vivo* assay to mimic the conditions of experiments with C. elegans. Pathogenicity of PA14 against C. elegans upon treatment with nafcillin or azlocillin and QQ lactonases is consistent with the trend observed in the PA14’s resistance profile against these compounds at 26°C (Fig. S16). QQ lactonase treatments increased the sensitivity of PA14 to nafcillin by up to 53% at 26°C (Fig. S16), and its virulence against C. elegans was also reduced in the presence of nafcillin (Fig. 7A). Similarly, the resistance of PA14 to azlocillin was increased by up to 15% upon treatment with QQ lactonases at 26°C (Fig. S16), and accordingly, its virulence against C. elegans was increased in the presence of QQ lactonases and azlocillin (Fig. 7C). Unexpectedly, at 26°C, unlike at 37°C (Fig. 4), lactonase and C4-HSL treatments failed to alter sulfathiazole sensitivity of PA14. Only 3oC12-HSL increased sulfathiazole sensitivity of PA14, by 74%. Strikingly, while the virulence of PA14 is also reduced by both lactonases as well as C4-HSL, it was increased by 3oC12-HSL in the presence of sulfathiazole (Fig. 7B), suggesting that the relationship between antibiotic resistance and its ability to kill the nematodes can be complex.

Taken together, these observations show that AHL-based QS and signal disruption can alter the pathogenicity of P. aeruginosa against C. elegans in the presence of antibiotics. QQ lactonases can modulate the sensitivity of PA14 to an antibiotic in a manner that may not always be reflected in changes in its virulence under similar conditions. Therefore, it appears that a combined administration of antibiotics and QQ agents can result in virulence changes that are difficult to predict and can prove to be either beneficial or detrimental to the host.

### Conclusion.

In this study, we demonstrate that the disruption of AHL-mediated QS signaling by the thermostable lactonases SsoPox and GcL can alter the antibiotic resistance profile of the Gram-negative pathogen P. aeruginosa PA14. This likely occurs via changes in the regulation of both intrinsic and adaptive resistance mechanisms. We were able to translate the observations from the Biolog Phenotype MicroArrays into experimental replicates using similar growth conditions for multiple antibiotics at their respective sublethal concentrations. Changes in antibiotic resistance against sulfathiazole and trimethoprim could be linked to key changes in the expression levels of genes involved in folate biosynthesis. Lastly, these observations were evaluated with an *in vivo* C. elegans infection model, and the results confirm that (i) the ability of P. aeruginosa to kill the nematode in the presence of certain antibiotics depends on QS, yet (ii) the combined effects of antibiotics and QQ lactonase are not always synergistic. Overall, whereas most previous studies investigating the effect of coadministration of QQ agents (QS inhibitors, enzymes) and antibiotics in bacterial infection models reported a positive synergistic effect (39, 46, 96), our results suggest that in P. aeruginosa, the relationship between antibiotics and QQ agents is very complex and depends on the type of antibiotic and substrate preference of the QQ agent used. More studies are needed, including on drug-resistant strains, to decipher the mechanisms of the synergies and antagonisms described in this work to assess and evaluate the potential of combination therapy in P. aeruginosa.

## MATERIALS AND METHODS

### Reagents, Pseudomonas strains, and growth conditions.

All bacterial strains are listed in Table S1. Pseudomonas aeruginosa strain UCBPP-PA14 (122), designated PA14 throughout this article, and its AHL synthase-deficient strains SM51 (PA14 Δ*lasI*), SM52 (PA14 Δ*rhlI*), and SM53 (PA14 Δ*lasI* Δ*rhlI*) (123) and the clinical isolate strain 19 (65), designated CI19 (obtained from Ryan Hunter, University of Minnesota Department of Microbiology and Immunology), were maintained in the laboratory following standardized protocols (124). PA14 was chosen because it is a reference PA strain. PA14 was cultured in either Miller’s Luria-Bertani (LB) broth (BD Difco, no. 244610), LB agar plates (BD Difco, no. 244510), or a peptone-based proprietary growth medium, GN IF-10A inoculating fluid (Biolog, no. 72264). CI19 was cultured in brain heart infusion (BHI) broth (BD Difco, no. 237500) or BHI agar instead of LB. Unless otherwise mentioned, all cultures were grown at 37°C with liquid cultures shaking at 250 rpm. All phenotype microarray plates—PM11C (no. 12211), PM12B (no. 12212), PM13B (no. 12213), PM14A (no. 12214), PM15B (no. 12215), PM16A (no. 12216), PM17A (no. 12217), PM18C (no. 12218), PM19 (no. 12219) and PM20B (no. 12220), and 100× dye mix A (no. 74221) were purchased from Biolog (Hayward, CA). *N-*butyryl-l-homoserine lactone, designated C4-HSL (no. 10007898), and *N*-3-oxo-dodecanoyl-l-homoserine lactone, designated as 3oC12-HSL (no. 10007895), were purchased from Cayman Chemical Company (Ann Arbor, MI) and dissolved in 100% dimethyl sulfoxide (DMSO) just before use. All other chemicals, including antibiotics, were of at least reagent grade and were purchased from either Millipore Sigma (Burlington, MA) or Fisher Scientific (Hampton, NH).

### Lactonase production, purification, and quantitation.

Production of the inactive SsoPox mutant 5A8 (125), SsoPox W263I (54), and GcL (58) in Escherichia coli strain BL21(DE3) was performed as previously described (53, 58, 68, 126). All lactonase preparations used in this study were made in buffer (PTE) composed of 50 mM HEPES, pH 8.0, 150 mM NaCl, and 0.2 mM CoCl_2_, sterilized by passing through a 0.2 μm-filter, and stored at 4°C until use.

### Preparation of antibiotic stocks.

Antibiotic stocks were prepared just before use by dissolution in deionized water (Nafcillin sodium, azlocillin sodium, colistin sulfate, sulfathiazole sodium, sulfadiazine sodium, d-cyclo-serine, carbenicillin disodium, and procaine hydrochloride), in 100% DMSO (oxacillin sodium, coumarin, trimethoprim, and carbonyl cyanide 3-chlorophenylhydrazone), in 0.1 N HCl (norfloxacin), or in 1 N NaOH (Ofloxacin). Antibiotic stocks were sterilized by passing through a 0.2-μm filter.

### Biolog Phenotype MicroArray experiments.

These experiments were conducted using a modification of a standard protocol obtained from Biolog, Inc. (Barry Bochner, direct communication). Phenotype MicroArray (PM) microplates allow for the testing of a range of antibiotics, inorganic salts, and bacteriostatic/bactericidal compounds (10 plates, labeled PM11 through PM20, each containing 24 compounds). Each tested compound is present at 4 concentrations, yet the precise concentration value is not disclosed by Biolog. Therefore, these MicroArrays provide a qualitative estimate of microbial sensitivity. The system is designed to use a proprietary peptone-based growth medium, GN IF-10A, to be supplemented with a proprietary tetrazolium redox dye mix. The dye is reduced to purple-colored formazan products (λ_abs_, 590 nm) due to NADH production (a sensitive indicator of respiration) by metabolically active cells (127, 128). This colorimetric reaction is monitored and recorded by a spectrophotometer at specific time intervals to generate a kinetic response curve mirroring microbial growth that can be further quantitated into parameters such as lag, slope, and area under the curve (69).

A single colony of PA14, picked from freshly streaked LB agar plates, was inoculated in 2 mL LB medium containing the appropriate treatments (50 μg/mL lactonases or *N*-acyl homoserine lactones at a final concentration of 10 μM) and grown at 37°C/250 rpm until cells reached the early log phase of growth (optical density at 600 nm [OD_600_], ~0.2 to 0.3). Cultures were then harvested by centrifugation (5, 000 × *g* for 3 min) and washed three times with Biolog IF-10A medium (1.2× GN IF-10A inoculating fluid diluted to 1× using autoclaved deionized water and containing 1× Biolog dye mix A). Washed cells were resuspended in Biolog IF-10A medium at an OD_750_ of ~0.05. A final inoculum solution was made by diluting the washed cells 1:100 into fresh Biolog IF-10A medium containing a similar treatment similar to that described above (50 μg/mL lactonases or *N*-acyl homoserine lactones at a final concentration of 10 μM). Then, 100 μL of the final inoculum solution was dispensed into each well of a 96-well Phenotype MicroArray microplate and overlaid with 20 μL sterile light mineral oil (Fujifilm Irvine, no. 9305) to limit evaporation. We note that the early addition of AHLs, at a low cell density, might lead to early activation of QS. Antibiotic-free growth of PA14 with 50 μg/mL inactive SsoPox 5A8 for control purposes was carried out on a half-area sterile 96-well microplate (Corning, no. 3696) to mimic the physical dimensions of Biolog PM microplates, using a protocol similar to that described above. The microplate was then incubated with a lid in a BioTek Epoch2 microplate spectrophotometer at 37°C with linear shaking at 300 rpm for 24 h. The OD_590_ (absorbance of formazan, as discussed earlier) and OD_750_ (for cell turbidity) were measured for each well every 15 min. The (OD_590_ – OD_750_) values plotted against the time of growth correspond to the growth curve of PA14, and the area under the curve (AUC), calculated with the BioTek Gen5 (v2.9) software corresponds to the total growth of PA14 over 24 h.

### Data analysis and presentation for the Biolog Phenotype MicroArray experiments.

Raw area-under-the-curve (AUC) data from the BioTek Gen5 software were analyzed and processed initially in Microsoft Excel worksheets before final analysis in GraphPad Prism 8. AUC values of less than 1,000 were found to be baseline noise and were discarded from subsequent analysis. Each antibiotic or antibacterial compound in the MicroArrays has 4 gradually increasing dosages (undisclosed by Biolog) in 4 horizontally adjacent microwells (denoted concentration 1 through concentration 4 in Fig. S1A and B to S5A and B), with the leftmost microwell containing the lowest dose (concentration 1) and the rightmost microwell containing the highest dose (concentration 4). The normalized growth allows us to compare treatments. It is the ratio between the AUC data for treatments (SsoPox W263I, GcL, C4-HSL, or 3oC12-HSL) and the AUC data for control (SsoPox 5A8; inactive lactonase), expressed in a percentage:

$$\text{Normalized Growth}\;\left(\text{\%}\right)=\frac{\text{AUC for PA14 growth with treatment}}{\text{AUC for PA14 growth with control}}\cdot 100$$

We present the treatment-dependent normalized results for all the 222 unique antibiotics and antibacterial compounds, organized according to their dosage (concentrations 1 to 4) in the form of graded heatmaps (white-red-black color schema) in Fig. S1 to S5. An additional heatmap strip with a green-black color scheme is overlaid on the right, which represents the sensitivity of PA14 to the indicated concentration of the compounds. The “relative resistance score” allows rapid comparison of the treatments. It is the ratio between the area-under-the-curve (AUC) of PA14 growth with the tested antimicrobial compound and the AUC of PA14 growth without the tested antimicrobial compound, expressed in a percentage:

$$\text{Relative Resistance Score}=\frac{\text{AUC of PA14 growth with compound}}{\text{AUC of PA14 growth without compound}}\cdot 100$$

A relative resistance score of 100% means that PA14 is completely resistant to the tested compound and that its growth is unaffected by the compound. For all measurements used for relative resistance calculation, the inactive lactonase 5A8 was used in all conditions (50 μg/mL). All heatmaps were generated and processed in GraphPad Prism 8. Shorter synonyms of the following compound names used by Biolog in the MicroArrays were obtained from PubChem (https://pubchem.ncbi.nlm.nih.gov/) and used in the heatmaps (Fig. 1 and 2 and Fig. S7) for esthetic purposes and are indicated in braces: methyltrioctylammonium chloride (methyltrioctyl-NH4Cl); 5,7-dichloro-8-hydroxyquinoline (chloroxine), sodium pyrophosphate decahydrate (sodium pyrophosphate); 1-chloro-2,4-dinitrobenzene (chlorodinitrobenzene); 5-nitro-2-furaldehyde semicarbazone (nitrofurazone); 1-hydroxypyridine-2-thione (pyrithione); 3, 4-dimethoxybenzyl alcohol (veratrole alcohol) and l-glutamic-g-hydroxamate (glutamine hydroxamate). The Biolog Phenotype MicroArray data (Fig. 1 and 2 and Fig. S1 to S7) represent one biological replicate that was used as a screen to identify key antimicrobial compounds for which resistance is likely to be regulated by QS. The identified compounds were subsequently validated in separate replicated experiments (Fig. 4 and Fig. S9 to S12).

### Screening of the data from the Biolog Phenotype MicroArray experiments.

Results from the MicroArrays (Fig. S1 to S5) were filtered, and this study focuses on compounds meeting all the following three criteria. (i) A differential change in PA14 growth is observed between the lactonase (SsoPox W263I and GcL) and AHL (C4-HSL and 3oC12-HSL) treatments at a given concentration. (ii) At this concentration, the antibiotic or antibacterial compound alters the growth of PA14 by at least 25% in at least one of the treatments (lactonase or AHL) compared to the control (5A8). (iii) The relative resistance score of PA14 against that antibiotic or antibacterial compound at that concentration should be 70% or less. These three filtering criteria eliminate compounds for which weak changes are observed and allow for the identification of molecules for which the resistance of PA14 is more likely to be regulated by QS signaling (Fig. 1 and 2). The first filter is self-explanatory, i.e., different outputs for QS and QQ treatment may mean that a biological pathway or mechanism regulated by QS is involved in the observed change in resistance. The second filter stems from the observation that changes below 25% did not robustly replicate in new culture experiments described in the following section. The third criterion is based on the fact that changes in resistance are more easily and robustly observed when the antibiotic treatment substantially reduces growth (Fig. S1 to S5).

### P. aeruginosa growth experiments with antibiotics.

We replicated key observations from the Phenotype MicroArray studies. For this purpose, we designed PA14 growth experiments using similar growth conditions on 96-well microplates. Growth, harvesting, and washing of PA14 were done similarly to the method described above. An inoculum solution is made by diluting the washed PA14 cells 1:100 into fresh Biolog IF-10A medium. Sublethal concentrations of all the following tested antibiotics and antibacterial compounds were determined by performing dose-response experiments using a growth protocol similar to the one described above (Fig. S8). The compounds that we tested included nafcillin (200 μg/mL), oxacillin (800 μg/mL), d-cyclo-serine (150 or 180 μg/mL), norfloxacin (0.25 μg/mL), ofloxacin (0.5 μg/mL), azlocillin (5 μg/mL), sulfadiazine (25 μg/mL), sulfathiazole (25 μg/mL), carbenicillin (50 μg/mL), trimethoprim (20 μg/mL), procaine (500 μg/mL), coumarin (500 μg/mL), carbonyl cyanide 3-chlorophenylhydrazone, or CCCP (50 μg/mL), and colistin (0.1 μg/mL). The growth of PA14 against these compounds at the indicated sublethal dosages in the presence of lactonases (SsoPox W263I or GcL, 50 μg/mL), inactive lactonase (SsoPox 5A8 mutant as the control, 50 μg/mL), or pure exogenously added AHLs (C4-HSL or 3oC12-HSL, 10 μM) was determined similarly to the Phenotype MicroArray experiments but conducted in replicates. Then, 150 μL of each set of supplemented inoculum solution was dispensed into wells of a 96-well sterile nonbinding polypropylene microplate (Corning, no. 3879) and topped with 50 μL sterile light mineral oil to prevent evaporation. The microplate was incubated, and the growth of PA14 was recorded in a BioTek Epoch2 microplate spectrophotometer as described above. For the replicated experiments at low growth temperature, PA14 was grown at 26°C (instead of 37°C) in the microplate reader for a duration of 48 h (instead of 24 h). For experiments with clinical strain CI19, it was grown in Biolog IF-10A medium like PA14, except that the preculture was started by inoculating a single colony from BHI agar plates into BHI broth instead of LB. Validation experiments performed with AHL synthase-deficient strains of PA14 were done in a similar manner as with PA14, described above, except that no lactonases or AHLs were added to these cultures.

### Total RNA extraction.

Overnight cultures of PA14 in LB medium were diluted 1:100 into fresh LB and grown until the OD_600_ was ~0.3 to 0.4. The cultures were harvested by centrifugation (5,000 × *g* for 3 min) and washed thrice with Biolog IF-10A medium (without dye mix A). Washed cells were resuspended in Biolog IF-10A medium (without dye mix A) at an OD_750_ of ~0.05. The washed cells were diluted 1:100 into 2 mL fresh Biolog IF-10A medium (without dye mix A) with or without sulfathiazole (25 μg/mL) or trimethoprim (20 μg/mL) in polypropylene culture tubes (Corning, no. 352059) and supplemented with 50 μg/mL enzymes or 10 μM (final) *N*-acyl homoserine lactones. Each condition was replicated 4 times. The cultures were incubated at 37°C for 22 h and shaken at 250 rpm. After incubation, the cultures were cooled on ice, and the cells from 1 mL of culture were harvested by centrifugation (6,000 × *g* for 5 min at 4°C). Cell pellets were frozen at −80°C until RNA extraction. Total RNA was extracted using the RNeasy minikit (Qiagen, no. 74104) following the manufacturer’s instructions. During purification, residual genomic DNA contamination was removed by an on-column DNase digestion performed using the RNase-free DNase I kit (Qiagen, no. 79254) following the manufacturer’s protocol at 37°C for 1 h. The quality and quantity of purified RNA were tested using Take3 plates on a BioTek Synergy HTX microplate reader. All extracted RNA was stored at −80°C until use.

### Quantitative reverse transcription PCR (qRT-PCR).

qRT-PCR was performed using the Power SYBR green RNA-to-CT 1-step kit (Thermo Fisher Scientific, no. 4389986) on a StepOnePlus real-time PCR (RT-PCR) system (Thermo Fisher Scientific) following the manufacturer’s instructions. RT-PCR-grade water (Ambion, no. AM9935) was used for setting up all reactions. Primers for PA14 *folA*, *folP*, and *recA* genes (Table S2) were initially characterized to determine optimal concentrations and efficiencies in qPCRs using the Thermo Fisher Scientific StepOne software (v2.3). Total reaction volumes were 10 μL. The primer concentrations used were 100 μM forward and reverse primers for *folA*, 200 μM forward and reverse primers for *folP*, and 100 μM forward and reverse primers for *recA*. The appropriate qRT-PCR controls—no template, no primer, and no reverse transcriptase controls—were performed for each reaction set. A relative quantification approach was undertaken to determine the amounts of *folA* and *folP* mRNA in each reaction, by using *recA* as the endogenous control gene.

### Caenorhabditis elegans liquid killing assays.

Some experimental conditions changes were required to adapt the PA14 antibiotic resistance study conditions to the C. elegans infection model, including the growth media and incubation temperature. The standardized liquid killing assay (129) requires a specific culture medium to coincubate the nematodes with PA14 at a temperature not exceeding 25°C, as that would otherwise be lethal for the nematodes. We noted that the Biolog IF-10A medium is toxic to C. elegans upon prolonged incubation but that the toxicity can be reduced significantly with dilution (data not shown). Therefore, a 1:1 dilution of IF-10A medium (without dye mix A) in the M9W buffer was chosen. It increases the mortality of C. elegans by 10 to 30% compared to a standard C. elegans maintenance buffer such as M9W in a 24- to 48-h incubation period (data not shown). We note that other buffer media, such as S-basal, S-complete, or SK medium (115) could not be used because they caused precipitation of the IF-10A medium upon mixing.

### Growth of C. elegans.

Strain SS104 [glp-4(bn2)] was obtained from the *Caenorhabditis* Genetics Center (CGC), University of Minnesota. The glp-4(bn2) mutation makes the nematodes incapable of producing offspring at temperatures greater than 20°C (130). This is necessary to prevent the formation of progeny nematodes during the incubation period, which would eventually hinder the downstream counting process. Nematodes were routinely maintained and cultured in the laboratory at 16°C on nematode growth medium (NGM) using standard protocols (115). We modified a liquid killing assay protocol (115, 129) for this study. Synchronization of SS104 nematodes was performed by hypochlorite isolation of eggs from gravid adults. The eggs were washed and hatched in M9W medium for 24 h at 23°C to generate starved L1 larvae. Larvae were collected by centrifugation (1, 000 × *g* for 1 min) and seeded on a 6-cm NGM agar plate with a lawn of E. coli strain OP50 as food for the nematodes. The plate was incubated at 23°C for 48 h to generate a synchronized population of young adult nematodes. The nematodes were washed off the plate using M9W, followed by 6 subsequent wash steps interspersed with gravity-mediated settling of nematodes, and finally suspended in M9W.

### Growth of bacteria.

Overnight cultures of E. coli OP50 and P. aeruginosa PA14 in LB medium were diluted 1:100 into fresh LB and grown until the OD_600_ was ~0.3 to 0.4. The cultures were harvested by centrifugation (5,000 × *g* for 3 min), washed thrice with M9W, and resuspended in M9W at a final OD_600_ of 0.3.

### Assay conditions.

Young adult SS104 nematodes were coincubated with P. aeruginosa PA14 in a 1:1 mix of IF-10A medium (without dye mix A) and M9W buffer for 24 h at 23°C with antibiotics (5 μg/mL azlocillin, 200 μg/mL nafcillin, or 25 μg/mL sulfathiazole) and lactonases (100 μg/mL) or AHLs (20 μM). All components of the assay were mixed in a sterile flat-bottom 96-well microplate (Sarstedt, no. 82.1581.001). Each well contained 200 μL of assay medium, composed of a mixture of M9W and Biolog IF-10A media (without dye mix A) at a ratio of 1:1 and supplemented with 10 μg/mL cholesterol, antibiotics, bacteria at a final OD_600_ of 0.03, and C. elegans (at least 20 to 50 young adult nematodes per well). For experiments with PA14 only, enzymes (100 μg/mL) or *N*-acyl homoserine lactones (20 μM) was also added. As a control experiment, young adult nematodes were incubated with E. coli OP50 in the same medium without antibiotics and lactonases/AHLs for a similar duration. OP50 is used as a standard food source for C. elegans during its routine laboratory maintenance and propagation, and therefore, the mortality observed when the nematodes are incubated with OP50 is treated as a control observation. For wells with OP50, sterile PTE buffer was added as a proxy for enzymes. Four replicates were performed for each condition that was tested. Due to the inherent variability in manually pipetting nematode suspensions (131), the number of nematodes dispensed in each well varied, but each well contained at least 20, and most wells contained 20 to 50 nematodes. The microplate was then sealed with gas-permeable Breathe-Easy membranes (Diversified BioTech, no. BEM-1) and incubated in a humidified incubator at 23°C for 24 h with shaking (300 rpm). After a 24-h coincubation period, the nematodes were allowed to settle at the bottom of the wells by gravity. Then, 100 μL of medium supernatant was discarded, and the remaining 100 μL containing the nematodes was transferred to unseeded 3.5-cm NGM agar plates. The plates were sealed with parafilm and incubated at 23°C for a 24-h recuperation period. The nematodes were then manually scored alive or dead under a microscope (Leica M165C) after touching them using a traditional worm pick (115).

### Data analysis.

The experimental data are represented as the percentage of nematodes that were alive after the entire incubation period for each treatment condition.

### Graphing and analysis of data.

All data processing, analysis, and subsequent graphing were done using either Microsoft Excel or GraphPad Prism 8. P. aeruginosa growth experiments were done in either 4 or 6 replicates. C. elegans and qPCR experiments were performed in quadruplicates. Rare, failed replicate measurements (e.g., absence of growth) were treated as outliers and were excluded from analysis. Unpaired two-tailed *t* tests with Welch’s correction were used for determining statistical significance and calculated using GraphPad Prism. All the raw experimental data and analysis for this study are available at https://doi.org/10.13020/1jgd-h107.
